# Prediction nomogram for evaluating the probability of postoperative fever in children with acute appendicitis

**DOI:** 10.3389/fped.2022.982614

**Published:** 2022-08-23

**Authors:** Yang Chen, Feng Ren, Dong Xiao, Ai-hui Guan, Le-dao Zhu, Xiao-peng Ma, Zhi-yong Wang

**Affiliations:** ^1^Shenzhen Children’s Hospital, Shenzhen, China; ^2^College of Medicine, Shantou University, Shantou, China

**Keywords:** postoperative fever, acute appendicitis, children, retrospective analysis, prediction model

## Abstract

**Objective:**

The purpose of this study was to establish a predictive model of postoperative fever in children with acute appendicitis through retrospective analysis, and the prediction ability of the model is demonstrated by model evaluation and external validation.

**Methods:**

Medical records information on children undergoing surgery for acute appendicitis within 2 years were retrospectively collected, prospective collection was performed for external validation in the next 3 months. The patients were divided into two groups according to whether the postoperative body temperature exceeded 38.5°C. Multivariate logistic regression analysis was used to determine independent risk factors and develop regression equations and nomogram. ROC curve, calibration curve and decision curve were made for model evaluation. Finally, the clinical implication of the prediction model was clarified by associating postoperative fever with prognosis.

**Results:**

High risk factors of postoperative fever included in the prediction model were onset time (X1), preoperative temperature (X2), leukocyte count (X3), C-reactive protein (X4) and operation time (X5). The regression equation is logit (P) = 0.005X1+0.166X2+0.056X3+0.004X4+0.005X5-9.042. ROC curve showed that the area under the curve (AUC) of the training set was 0.660 (0.621, 0.699), and the AUC of the verification set was 0.712 (0.639, 0.784). The calibration curve suggested that the prediction probability was close to the actual probability. Decision curve analysis (DCA) showed that patients could benefit from clinician’s judgment. Furthermore, prognostic analysis showed children presenting with postoperative fever had the more duration of postoperative fever, hospitalization stays and cost, except for rehospitalization.

**Conclusion:**

All the results revealed that the model had good predictive ability. Pediatricians can calculate the probability of postoperative fever and make timely interventions to reduce pain for children and parents.

## Introduction

Acute appendicitis is a common cause of acute abdominal pain in children that usually necessitates surgery (1). Postoperative fever is the most frequent condition of appendectomy, primarily caused by the gradual absorption of inflammatory exudate and abdominal hemorrhage, but also by residual or recurrent infection (2). Suspected infections and additional treatment will undoubtedly result in more hospital stays and costs. Meanwhile, the elevated temperature causes a decrease in spirit and appetite, which exacerbates children’s suffering and parental anxiety (3, 4).

Many studies have shown that postoperative fever in children with appendicitis is mainly associated with residual abdominal inflammation and incisional infection, both of which cause fever usually higher than 38.5°C, and this temperature is also indicative of the antipyretic’s application (5). However, in addition to inflammatory factors, surgical stress and individual reactions impact postoperative fever in appendicitis, and the temperature level may vary in different individuals. Early identification of risk factors for postoperative fever in children with appendicitis can help clinicians to predict and intervene early, including prolonging or increasing the application of antibiotics and improving surgical incision care (6, 7).

Therefore, this study was conducted to investigate the risk factors affecting postoperative fever in children with appendicitis by analyzing a large number of medical records of children with surgical treatment of acute appendicitis and developing a predictive model for clinical work.

## Materials and methods

### Patients

This study was approved by the Ethics Committee of Shenzhen Children’s Hospital (No. 2021059). The information on cases of children under 14 years old who underwent surgery for acute appendicitis from January 1, 2020, to January 1, 2022, in Shenzhen Children’s Hospital was retrospectively collected as the training set; the information on cases of acute appendicitis in children from January 1 to March 1, 2022, was prospectively collected as the validation set for external validation.

All included cases were operated by laparoscopic appendectomy and diagnosed with appendicitis by surgery and postoperative pathology. Antibiotics were given preoperatively and postoperatively, and appropriate rehydration was given after surgery according to the food intake. Since laparoscopic minimally invasive surgery is now widely used, postoperative analgesia is not routine. Ibuprofen is used to reduce fever when the postoperative temperature is higher than 38.5°C, and if the temperature does not decrease significantly, acetaminophen may be added.

The exclusion criteria included cases that had received relevant treatment prior to admission, cases diagnosed with chronic appendicitis, cases with other intestinal diseases in combination, cases with a temperature not reaching 38.5°C using antipyretic drugs, cases with concurrent respiratory or urinary infections, and cases with incomplete information.

### Information collection

The children with postoperative body temperature greater than 38.5°C were classified as the fever group, otherwise as the non-fever group. According to the most reliable contents in the medical records, age, height, weight, clinical symptoms, onset time (from symptom expression to surgery), laboratory examination and ultrasound imaging characteristics at admission, preoperative temperature, operation time, and pathological manifestations were collected. Duration of postoperative fever, hospitalization stays and cost, whether or not readmission because of appendicitis complications were considered as prognosis.

### Statistical methods

SPSS26.0 and R software (version 3.1.1) were performed for statistical analysis. Independent sample *T*-test, Kruskal-Wallis, and Chi-square test were used to comprehensively summarize the clinical characteristics of the enrolled children with appendicitis. The data conforming to normal distribution were expressed as mean ± standard deviation, otherwise median ± quartile, and categorical variables were illustrated by proportion. Univariate logistic regression was performed to initially screen out potential variables. Then, a multivariable logistic regression analysis was performed to identify the risk factors closely associated with postoperative fever. In addition, regression equations were calculated, and nomograms were plotted. Furthermore, the ROC curve and calibration curve were plotted from the data of the training set and validation set for model evaluation and external verification. The decision curves were plotted by calculating the net benefits corresponding to different threshold probabilities to evaluate the application value of the model. Finally, the correlation between postoperative fever and prognosis was analyzed to explore the clinical significance of the prediction model.

## Results

### General information

A total of 1,001 eligible children were included in this study as the training set, and 234 children were included in the validation set. The clinical information of the fever and non-fever groups in the training set is presented in Table 1. Baseline information such as gender, age and BMI were not statistically significant between the two groups (*P* > 0.05).

**TABLE 1:** Comparison of clinical characteristics between the two groups.

Variates	Non-fever (*n* = 737)	Fever (*n* = 264)	χ ^2^/t	*p*
**Baseline information**				
Gender (male/female)	467/270	152/112	2.761	0.097
Age/years	8.11 ± 2.87	8.11 ± 3.11	–0.011	0.991
BMI	17.31 ± 3.66	17.33 ± 3.82	–0.085	0.932
**Clinical information**				
Onset time/hours	35.89 ± 34.17	47.4 ± 37.48	–4.576	< 0.001
Vomiting (yes/no)	427/310	168/96	2.618	0.106
Abdominal tension (yes/no)	257/480	107/157	2.69	0.101
Leukocytes count/ × 10^9^/L	16.08 ± 4.71	17.53 ± 5.63	–4.051	< 0.001
Neutrophil ratio/%	82.88 ± 9.35	84.56 ± 8.68	–2.547	0.011
Eosinophils count/ × 10^9^/L	0.331 ± 1.994	0.198 ± 0.700	1.058	0.29
C-reactive protein/mg/L	40.06 ± 47.36	61.46 ± 64.45	–5.681	< 0.001
Platelets/ × 10^9^/L	312.11 ± 185.56	307.88 ± 80.70	0.357	0.721
Total bilirubin/mg/dL	13.39 ± 8.33	14.75 ± 7.87	–2.301	0.022
Appendix diameter/mm	10.36 ± 3.25	10.57 ± 2.75	–0.924	0.356
Hypoecho (yes/no)	248/489	116/148	8.808	0.003
Preoperative temperature/?	37.91 ± 0.90	38.18 ± 0.99	–4.001	< 0.001
Operation time/min	56.57 ± 27.74	65.51 ± 31.74	–4.321	< 0.001
**Prognosis**				
duration of fever/hours	18.96 ± 24.62	31.35 ± 35.95	–6.159	< 0.001
Hospital stays/days	7.07 ± 2.80	7.97 ± 2.96	–4.401	< 0.001
Hospital cost/¥	13785.5 ± 3563.4	14592.8 ± 3984.9	–3.059	0.002
Rehospitalization (yes/no)	31/706	18/246	2.848	0.091

### Variables screening

Univariate logistic regression analysis was performed for all variables, and *P*-values and effect for each variable are shown in Table 2. Variables such as vomiting, abdominal tension, neutrophil ratio, eosinophils, platelets, and appendix diameter were excluded (*p* > 0.2).

**TABLE 2:** Univariate logistic regression for variables filtration.

Variates	β	P	Exp (B) (Cl%95)
Onset time	0.006	0.006	1.006 (1.002–1.011)
Vomiting	0.089	0.574	1.093 (0.801–1.494)
Abdominal tension	–0.025	0.878	0.976 (0.711–1.339)
Leukocytes count	0.051	0.002	1.052 (1.019–1.086)
Neutrophil ratio	0.006	0.544	1.006 (0.986–1.028)
Eosinophils count	–0.044	0.473	0.957 (0.847–1.080)
C-reactive protein	0.004	0.009	1.004 (1.001–1.007)
Platelets	0.001	0.503	1.000 (0.998–1.001)
Total bilirubin	0.013	0.151	1.013 (0.995–1.030)
Appendix diameter	–0.010	0.679	0.990 (0.942–1.040)
Hypoecho	0.239	0.131	1.269 (0.932-1.730)
Preoperative temperature	0.137	0.102	1.146 (0.973–1.351)
Operation time	0.005	0.044	1.005 (1.000–1.011)

### Model establishment

**FIGURE 1:**
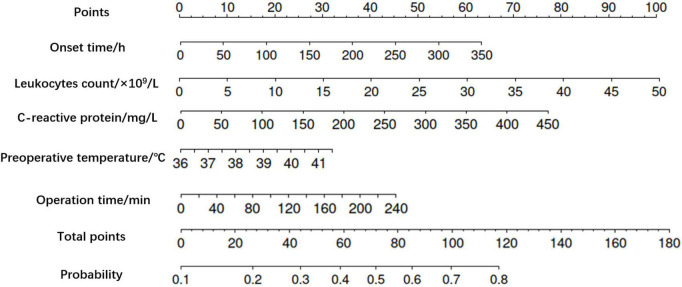
Nomogram of postoperative fever in children with acute appendicitis. The higher the total score calculated according to the predictors, the higher the probability of postoperative fever.

According to the results in Table 2, the preliminary screened variables were incorporated into the multivariate regression model, and the variables that were finally determined into the model were onset time, preoperative temperature, leukocyte count, C-reactive protein and operation time. The regression equation is logit (P) = 0.005X_1_ + 0.166X_2_ + 0.056X_3_ + 0.004X_4_ + 0.005X_5_−9.042, where X_1_ was onset time (hour), X_2_ is preoperative temperature (°C), X_3_ was leukocyte count (× 10^9^/L), X_4_ was C-reactive protein (mg/L), X_5_ was operation time (min). See more details in Table 3. Moreover, the more practical nomogram representing the regression equation was shown in Figure 1.

**TABLE 3:** Multivariate logistic regression for predictive equation.

Variates	β	Standard errors	Wald	*P*	Exp (B) (Cl%95)
Onset time	0.005	0.002	6.692	0.010	1.005 (1.001–1.010)
Leukocytes count	0.056	0.015	14.567	< 0.001	1.058 (1.028–1.089)
C-reactive protein	0.004	0.001	8.763	0.003	1.004 (1.001–1.007)
Preoperative temperature	0.166	0.081	4.233	0.040	1.181 (1.008–1.384)
Operation time	0.005	0.003	4.051	0.044	1.005 (1.000–1.010)
Constant	–9.042	3.049	8.796	0.003	

### Model evaluation

**FIGURE 2:**
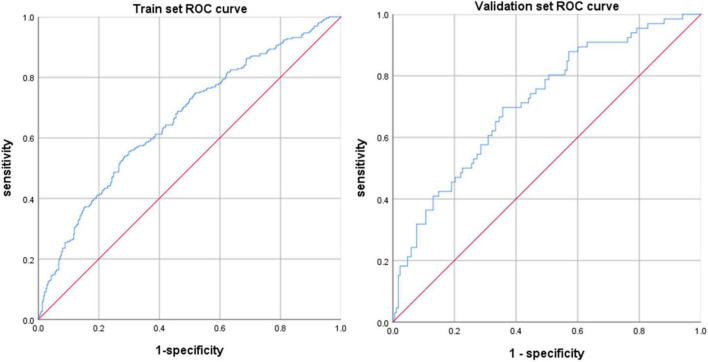
ROC curve for the train set and validation set. The prediction ability still had good performance in validation set, which indicated that the prediction model obtained through the training set had not been overfitted.

**FIGURE 3:**
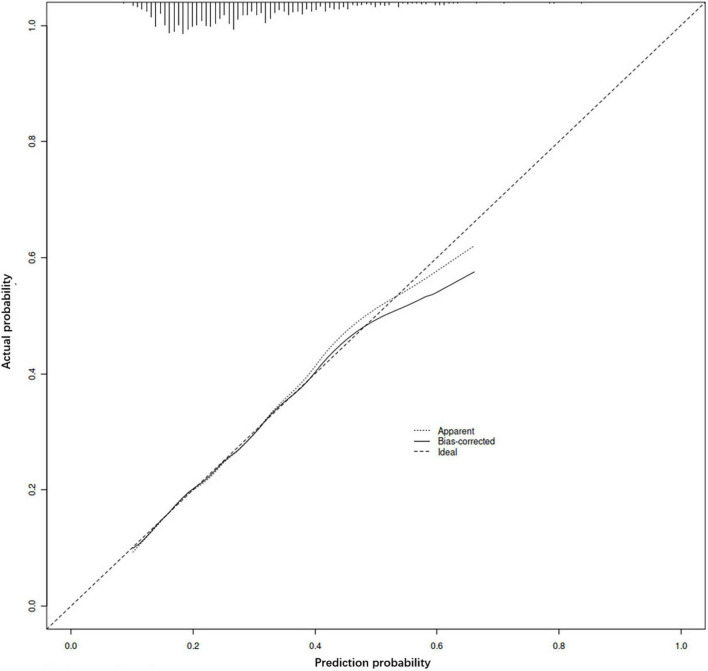
Calibration curve of the prediction model. The accuracy of the model is judged by observing the coincidence between the prediction probability of random sampling and the actual situation.

**FIGURE 4:**
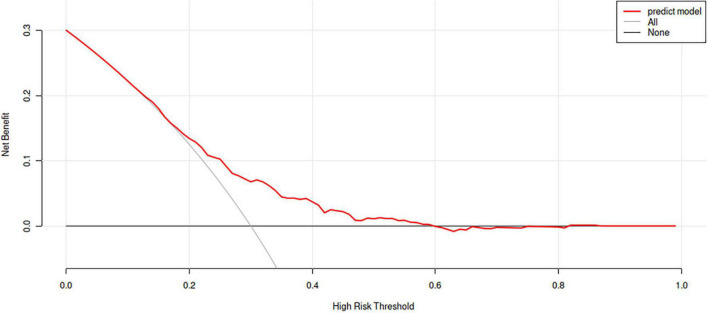
Decision curve analysis for the prediction model. The horizontal line represents the net benefit without any intervention, and the oblique line represents the net benefit of intervention at all the threshold probability. The further away the curve is from these two lines, the greater benefit patients gain from the predicted results.

The ROC curves were drawn by calculating the prediction probability according to the regression equation for the training set and the verification set respectively (Figure 2). The area under the curve (AUC) of the training set was 0.660 (0.621, 0.699), and the AUC of the verification set was 0.712 (0.639, 0.784). Meanwhile, the calibration curve (Figure 3) suggested that the curves of random sampling in the training set were close to the ideal line. The Hosmer–Lemeshow test for the training set indicated that the Chi-square value was 5.234, *p* = 0.732 > 0.05, which proved that the prediction model had no obvious over-fitting. Decision curve analysis (DCA) describing decision-making proceeds showed there was an acceptable range of threshold probability that patients could benefit from clinician’s judgment (Figure 4). The above results revealed that the prediction model had good performance.

## Discussion

Postoperative fever of appendicitis is mainly caused by inflammatory response and resorption, with a small part of it is attributed to secondary infection (8). Fever in the early postoperative phase for a short duration is a normal manifestation due to the natural absorption of hemorrhage and exudation. The immune response caused by surgical trauma and the gradual reduction of inflammation in the body all have an effect on fever (8, 9). Physiological fever after surgery has a thermal peak that is usually less than 38.5°C and remains for about 2–3 days. However, infection can stimulate immune cells to secrete inflammatory factors that raise the temperature setting, especially in more severe incisional infections and inflammation of the appendiceal stump, in which the temperature usually remains elevated and tends to exceed 38.5°C (10, 11).

There are few studies on predictive models for postoperative fever in children with appendicitis. Most of the data in previous studies came from daily temperature measurements and there were many cases of NSAID application for pain relief, which can obscure the febrile manifestations in some of the children (12, 13). In this study, on the other hand, temperature recording at four-hourly intervals and rigorous documentation of antipyretic application could provide a more accurate description of postoperative fever. The treatment and care measures of acute appendicitis have been standardized as a clinical pathway disease in our hospital since 2010. Therefore, the differences in treatment and nursing measures among all cases in the data included in this study were minor, and the resulting postoperative fever prediction model was more consistent with clinical realities. Pediatricians can predict the probability of post-appendectomy fever from a simple history and relevant examination findings, so that early interventions can be made to reduce the suffering and hospitalization cost of children.

Statistical analysis in this study showed that the onset time, preoperative temperature, leukocyte count, C-reactive protein, and operation time were independent risk factors for postoperative fever in children with acute appendicitis. The differentiation and calibration of the prediction model were satisfactory. The higher the prediction probability calculated by the regression equation and the nomogram, the greater the likelihood of postoperative temperature exceeding 38.5°C. Also, the higher the temperature, the more severe the infection (12, 13). Then, further analysis of the prognosis of both groups revealed that the duration of postoperative fever, postoperative hospital stays, and costs were significantly higher in the fever group than in the non-fever group (*P* < 0.5). However, there was no significant correlation between postoperative fever and readmission rate due to complications of appendicitis (*P* > 0.5), which suggests that postoperative readmission may be related to other factors and needs further study. These findings are consistent with clinical practice.

Delayed treatment often leads to a more serious condition, which makes the mucosal barrier of the appendix more vulnerable to bacterial invasion (14). The preoperative temperature mirrors the systemic inflammatory response, and although the primary lesion has been surgically removed, this impact does not disappear immediately (13). In addition, exposure because of prolonged operation increases the risk of infection, while anesthesia and sedation compromise the immune system (15). Leukocytes and C-reactive protein have been shown to be reactive indicators of infection and inflammation (16), while platelets, eosinophils, and bilirubin are significantly altered during severe infection (17), which may be the reason for their exclusion from the model. Similarly, the proportion of neutrophils representing bacterial infection was not included. The Pearson calculation between neutrophil ratio and leukocytes showed a strong correlation (*r* = 0.843, *P* < 0.001), which may make the neutrophil ratio covered for avoiding collinearity because appendicitis results from bacterial infection primarily. Besides, vomiting and abdominal tension have been controversial because of inaccurate medical records and lack of cooperation from children (18). Appendix tumefaction and hypoechoic are important performances for diagnosing appendicitis by ultrasonography (19). However, the diameter of the appendix increases with the age of children, and mucosal injury is influenced by the size, shape, and location of fecalith, which confounds the association between the ultrasound presentation and the severity of the infection (20).

We developed a clinical prediction model by fully considering demographic characteristics, clinical presentation, laboratory tests, imaging performance, and surgical information, and excluded subjective indicators whenever possible. Moreover, external validation was performed by prospectively collecting new cases to prevent overfitting. Further multicenter experiments are needed to validate the model in the future.

## Conclusion

Postoperative fever in children with acute appendicitis is closely associated with the length of onset, leukocytes count, C-reactive protein, preoperative temperature, and duration of surgery, and pediatric surgeons can assess the probability of postoperative fever using this predictive model in order to make appropriate treatment strategies and early interventions.

## Data availability statement

The original contributions presented in this study are included in the article/Supplementary material, further inquiries can be directed to the corresponding author/s.

## Ethics statement

The studies involving human participants were reviewed and approved by the Medical Ethics Committee of Shenzhen Children’s Hospital. Written informed consent to participate in this study was provided by the participants or their legal guardian/next of kin.

## Author contributions

YC and FR together completed data collection and statistical analysis. YC was responsible for manuscript writing and submitting. Z-YW and X-PM designed the whole process of the study and participated in the modification of the manuscript. DX, A-HG, and L-DZ contributed to the data organization and article revision. All authors contributed to the article and approved the submitted version.
